# PHYSICAL ACTIVITY AMONG OLDER ADULTS WITH AND WITHOUT HIV IN UGANDA: COHORT STUDY IN RURAL UGANDA

**DOI:** 10.1093/geroni/igae098.1925

**Published:** 2024-12-31

**Authors:** Aneeka Ratnayake, Yao Tong, Zahra Reynolds, Steffany Chamut, Robert Paul, Jeremy Tanner, Brianne Olivieri-Mui, Mark Siedner

**Affiliations:** Roux Institute at Northeastern University, Portland, Maine, United States; Harvard University, Cambridge, Massachusetts, United States; Massachusetts General Hospital, Boston, Massachusetts, United States; Harvard School of Dental Medicine, Boston, Massachusetts, United States; University of Missouri St. Louis, St. Louis, Missouri, United States; The University of Texas Health San Antonio, San Antonio, Texas, United States; Northeastern University, Boston, Massachusetts, United States; Harvard University, Cambridge, Massachusetts, United States

## Abstract

Studies from high-income countries have established the benefits of physical activity (PA) for health among older people. Yet, PA and its links to frailty and quality of life (QOL) in older rural African populations living with HIV remain under-explored. We analyzed data from the first three annual assessments in a cohort of older people living with HIV (PLWH) and age- and sex-similar, community-based people not living with HIV. PA was measured through self-reported minutes of moderate and vigorous PA, converted into metabolic equivalent of task (METs). PA adherence to World Health Organization (WHO) guidelines was defined as a minimum of 150 minutes of moderate PA or 75 minutes of vigorous PA weekly. Linear and logistic generalized estimating equations (GEE) regression models were employed to estimate the association between HIV status and PA, adjusting for frailty, QOL, comorbidities, and age. At enrollment there were 297 PLWH and 302 people not living with HIV, 304 men and 295 women, with a median age of 58 years (IQR:54-63); 96% met PA guidance. After multivariable adjustment, PA in METs was associated with younger age (b=-187.18 per year, p<0.0001), HIV infection (b=-1039.12, p=0.041), higher health-related QOL (b=38.05 per point on a 0-100 scale, p=0.034), frailty (b=-2661.24, p=0.028), and medical comorbidity (b=-2208.07, p<0.0001). Guideline-concordant PA was inversely associated with frailty (OR:0.27, p=0.0076) and medical comorbidity (OR:0.43, p=0.0069). Correlates of lower PA were older age, living with HIV, frailty, and medical comorbidity. Those with frailty and medical comorbidity may not be benefitting from recommended levels of PA.

